# *Escherichia coli* O157:H7 bacteriophage Φ241 isolated from an industrial cucumber fermentation at high acidity and salinity

**DOI:** 10.3389/fmicb.2015.00067

**Published:** 2015-02-17

**Authors:** Zhongjing Lu, Fred Breidt

**Affiliations:** ^1^Department of Molecular and Cellular Biology, Kennesaw State UniversityKennesaw, GA, USA; ^2^USDA Agricultural Research Service – Department of Food, Bioprocessing, and Nutrition Sciences, North Carolina State UniversityRaleigh, NC, USA

**Keywords:** bacteriophage, phage Φ241, *Escherichia coli* O157:H7, biocontrol agent, cucumber fermentation, high acidity and salinity, food safety

## Abstract

A novel phage, Φ241, specific for *Escherichia coli* O157:H7 was isolated from an industrial cucumber fermentation where both acidity (pH ≤ 3.7) and salinity (≥5% NaCl) were high. The phage belongs to the *Myoviridae* family. Its latent period was 15 min and average burst size was 53 phage particles per infected cell. The phage was able to lyse 48 *E. coli* O157:H7 strains, but none of the 18 non-O157 strains (including *E. coli* O104:H7) or the 2 O antigen-negative mutants of O157:H7 strain, 43895Δ*per* (also lacking H7 antigen) and F12 (still expressing H7 antigen). However, the phage was able to lyse a *per*-complemented strain (43895Δ*per*Comp) which expresses O157 antigen. These results indicated that phage Φ241 is specific for O157 antigen, and *E. coli* strains lacking O157 antigen were resistant to the phage infection, regardless of the presence or absence of H7 antigen. SDS-PAGE profile revealed at least 13 structural proteins of the phage. The phage DNA was resistant to many commonly used restriction endonucleases, suggesting the presence of modified nucleotides in the phage genome. At the multiplicity of infection of 10, 3, or 0.3, the phage caused a rapid cell lysis within 1 or 2 h, resulting in 3.5- or 4.5-log-unit reduction in cell concentration. The high lytic activity, specificity and tolerance to low pH and high salinity make phage Φ241 a potentially ideal biocontrol agent of *E. coli* O157:H7 in various foods. To our knowledge, this is the first report on *E. coli* O157:H7 phage isolated from high acidity and salinity environment.

## INTRODUCTION

*Escherichia coli* O157:H7 has emerged as one of the major food-borne pathogens. Each year, it causes more than 73,000 illnesses, 2,100 hospitalizations, and 60 deaths in the U.S. (Mead and Griffin, 1998; Mead et al., 1999; Rangel et al., 2005). A variety of foods have been associated with these outbreaks such as undercooked ground beef (Griffin and Tauxe, 1991; Anonymous, 1993, 2014; Bell et al., 1994), raw milk (Riley et al., 1983), cheese (Anonymous, 2010), bologna (Anonymous, 2011), cold sandwiches (Karmali, 1989), water (Swerdlow et al., 1992; Bopp et al., 2003), unpasteurized apple juice (Anonymous, 1996), sprouts, lettuce, spinach, and other vegetables (Como-Sebetti et al., 1997; Jinneman et al., 2003; Anonymous, 2006, 2012a,b, 2013). Healthy cattle are the primary reservoir of *E. coli* O157:H7. Human infection by *E. coli* O157:H7 can frequently be traced to the food or water contaminated with cattle manure (Gyles, 2007). The infection by this pathogen can result in severe hemorrhagic colitis and life-threatening hemolytic uremic syndrome (Remis et al., 1984; Cleary, 1988; Tarr, 1995; Nataro and Kaper, 1998). *E*. *coli* O157:H7 has a very low infectious dose (as low as 10 cells; Griffin and Tauxe, 1991; Griffin et al., 1994; Tuttle et al., 1999) partly due to its very efficient mechanisms of stress resistance (Price et al., 2004). Acid resistance is one of the characteristics of *E*. *coli* O157:H7. The bacterium has evolved multiple mechanisms to survive in low-pH environments (Lin et al., 1996; Castanie-Cornet et al., 1999; Jordan et al., 1999; Price et al., 2000, 2004; Large et al., 2005) such as gastrointestinal tracts and various acidic foods (Weagant et al., 1994; Diez-Gonzalez and Russell, 1999; Price et al., 2004). Acid resistance is especially crucial for food-borne pathogens that must survive the hostile acidic condition in the stomach before entering and colonizing the small intestines or colon (Berk et al., 2005; Chen and Jiang, 2014).

Acid adaptation can further enhance the survival of *E. coli* O157:H7 in fermented or acidified foods, and induce the cross-protection against heat, salt, and acids (Farber and Pagotto, 1992; Leyer and Johnson, 1993; Leyer et al., 1995; Cheville et al., 1996). A variety of acidic foods have been involved in the outbreaks caused by *E. coli* O157:H7. These include apple cider (Besser et al., 1993; Hilborn et al., 2000), unpasteurized apple juice (Cody et al., 1999), salami (Anonymous, 1995), and fermented sausage (Glass et al., 1992). *E. coli* O157:H7 can also tolerate high concentration of NaCl (Glass et al., 1992).

Many physical, chemical, and biological methods (such as pasteurization, radiation, addition of preservatives, or addition of lactic acid bacteria) have been used to control *E. coli* O157:H7 in foods. However, these control methods are not very effective for certain foods or they can alter the color, flavor, or texture of the foods. Safe and effective alternative methods are needed to control *E. coli* O157:H7 in foods. Recent studies have showed that the use of phages to control pathogenic bacteria in foods is a promising novel strategy.

The use of phages as antibacterial agents has several advantages over traditional antibacterial methods. First of all, phages are highly host specific. They only infect specific bacterial hosts and cause rapid bacterial lysis. They do not infect humans and other eukaryotes. Phages specific for pathogenic bacteria do not disrupt normal microflora in humans (Kudva et al., 1999) or in animals. Secondly, phages are not toxic to humans. Although certain cell lysis may release endotoxins, phages themselves do not generate any toxic products during their multiplication (Hagens and Loessner, 2010). Thirdly, phages do not alter food quality because they do not produce any substances that can change the taste, composition, aroma, or color of foods. In addition, phages are stable (Coffey et al., 2010), but also self-limiting in foods. They do not replicate unless their bacterial hosts are present (Hagens and Loessner, 2010). Moreover, phages are the most abundant biological entities and naturally present in the environment and a wide variety of foods (Guenther et al., 2009). It is relatively easy to isolate phages from the environment and propagate them in laboratories. All these features make phages promising novel biocontrol agents of bacterial pathogens in foods.

Recent studies have shown high efficacy of using phages against several major food-borne pathogens including *E*. *coli* O157:H7, *Listeria monocytogenes*, and *Salmonella enterica* in food products or on food contact surfaces. Use of phages specific for *E. coli* O157:H7 resulted in significant, log-unit reductions in *E. coli* O157:H7 counts in a variety of foods such as tomato, spinach, broccoli, and ground beef (Abuladze et al., 2008), beef (Carter et al., 2012), cantaloupe (Sharma et al., 2009), lettuce (Sharma et al., 2009; Ferguson et al., 2013), and other leafy green vegetables (Viazis et al., 2011). Such reductions could substantially decrease a risk of food-borne infections by the pathogen.

Significant progress in phage research for food safety has been made toward phage applications in foods. Several phage-based food additives have been recently approved or cleared by the U.S. Food and Drug Administration (FDA). These approvals have increased the impetus of phage research to uncover phage-mediated applications against other food-borne pathogens (Mahony et al., 2011). It is likely that more phage products will be developed and gradually gain market acceptance by the food industry and the consumers as a means of a safe, natural, and effective prevention of food-borne diseases (O’Flaherty et al., 2009; Sharma, 2013).

Phages specific for *E. coli* O157 have previously been isolated from human fecal materials or animal manures from bovine, ovine, swine, and chicken (Kudva et al., 1999; Morita et al., 2002; O’Flynn et al., 2004; Tomat et al., 2013), lake or pond water (Shahrbabak et al., 2013), and sewage (Sheng et al., 2006; Shahrbabak et al., 2013). No *E. coli* O157-specific phages were isolated from the environment where both acidity and salinity are high. The objectives of this study were to isolate an *E. coli* O157:H7-specific phage from a cucumber fermentation with low pH (3.7) and high salt concentration (5% NaCl), to characterize the phage, and to evaluate the potential of the phage as an effective biocontrol agent against *E. coli* O157:H7 in various foods.

## MATERIALS AND METHODS

### BACTERIAL STRAINS AND CULTURE CONDITIONS

The *E. coli* strains used in this study are listed in **Tables 1** and **2**. A total of 46 *E. coli* O157:H7 strains, and 18 *E. coli* non-O157:H7 strains from various sources were obtained from the culture collection of USDA Agricultural Research Service located at North Carolina State University. The non-O157 strains included a variety of *E. coli* strains that express a variety of H antigens including H7 antigen. Two previously described O antigen-negative mutants (43895Δ*per* and F12), one *per*-complemented mutant (43895Δ*per*Comp), and two *E. coli* O157:H7 parent strains (ATCC 43895 and 8624) were kindly provided by Pina Fratamico (**Table 3**). All strains were stored in tryptic soy broth (TSB; Difco) supplemented with 16% (v/v) glycerol at -80°C until use. Fresh overnight culture of each *E. coli* strain was prepared by inoculating 10 ml of TSB with an isolated colony from a tryptic soy agar (TSA) plate and incubating statically for 12 h at 37°C. For phage lysate preparation, TSB broth was supplemented with 10 mM CaCl_2_ (Sigma–Aldrich, St. Louis, MO, USA) unless otherwise stated. Soft TSA agar used in plaque assay was prepared with TSB broth supplemented with 0.6% agar.

**Table 1:** *Escherichia coli* O157:H7 strains that are sensitive to phage Φ241.

ID^a^	Serotype	Source
**B0201**^**b**^	O157:H7	Apple cider outbreak
**B0349**	O157:H7	Spinach outbreak
B0264	O157:H7	Apple juice outbreak, 1996
**B0204**	O157:H7	Pork
**B0202**	O157:H7	Salami outbreak
B0203	O157:H7	Ground beef
B0348	O157:H7	Salami
B0350	O157:H7	Sakai
B0243	O157:H7	Bovine carcass
B0242	O157:H7	Bovine carcass
B0240	O157:H7	Bovine carcass
B0239	O157:H7	Bovine carcass
B0238	O157:H7	Bovine carcass
**B0241**	O157:H7	Bovine carcass
B0258	O157:H7	Bovine feces
B0259	O157:H7	Bovine feces
B0301	O157:H7	Water
B0307	O157:H7	Water
B0306	O157:H7	Water
B0309	O157:H7	Water
B0302	O157:H7	Water
B0297	O157:H7	Water
B0299	O157:H7	Water
B0285	O157:H7	Water
B0275	O157:H7	Water
B0305	O157:H7	Water
B0281	O157:H7	Water
B0289	O157:H7	Water
B0280	O157:H7	Water
B0287	O157:H7	Water
B0283	O157:H7	Water
B0269	O157:H7	Human, outbreak, 2000, waterborne
**B0273**	O157:H7	Human, outbreak, 2002, leafy vegetable
B0247	O157:H7	Human, outbreak
**B0296**	O157:H7	Human, outbreak, 2005, leafy vegetable
**B0311**	O157:H7	Human, outbreak, 2006, leafy vegetable
B0246	O157:H7	Human, outbreak
**B0271**	O157:H7	Human, outbreak, 2003, leafy vegetable
B0250	O157:H7	Human, outbreak
B0263	O157:H7	Human, sporadic, 1997,
B0251	O157:H7	Human, outbreak
B0249	O157:H7	Human, outbreak
B0266	O157:H7	Human, outbreak, 1999, taco meat
B0245	O157:H7	Human, outbreak
**B0265**	O157:H7	Human, outbreak, 1999, lettuce
B0244	O157:H7	Human, outbreak

*^a^ID, identification number in the culture collection of USDA-ARS Food Fermentation Laboratory*.

*^b^The strains with ID bolded were used for initial phage isolation*.

**Table 2:** Non-O157 strains of *E. coli* that are resistant to phage Φ241.

ID^a^	Serotype	Source
B0445	O26:H11	Human
B0449	O26:H11	Human
B0463	O103:H6	Human diarrhea
B0460	O103:H25	Human
B0469	O104:H4	Human
B0467	O104:H21	Human, milk outbreak
B0475	O111:NM^b^	Human
B0478	O111:H8	Human
B0479	O121:NM	Human diarrhea
B0485	O145:NM	Human
B0457	O45:H2	Cow (calf)
B0468	O104:H7	Ground beef
B0235	Non-O157^c^	Bovine feces
B0237	Non-O157	Bovine feces
B0234	Non-O157	Bovine feces
B0236	Non-O157	Bovine feces
B0233	Non-O157	Bovine feces
25922	O6:H1	ATCC^d^

*^a^ID, identification number*

*^b^NM, non-motile*.

*^c^The strains were not completely serotyped. But the data showed that they did not respond to the serum antibody against O157 strains*

*^d^ATCC, American Type Culture Collection*.

**Table 3:** Phage susceptibility of *E. coli* O157:H7 strains and their O antigen-negative mutants.

*E. coli* strain	Description	Plaque formation^a^	Source or reference
ATCC 43895	Wild-type *E. coli* O157:H7, clinical isolate, stx_1_^+^/stx_2_^+^	+	ATCC^b^
43895Δ*per*	O antigen-negative mutant of ATCC 43895 with perosamine synthetase deleted	–	Sheng et al. (2008)
43895Δ*per*Comp	43895Δ*per* transformed with pCRII::per	+	Sheng et al. (2008)
8624	Wild-type *E. coli* O157:H7, clinical isolate, stx_1_/stx_2_^+^	+	Bilge et al. (1996)
F12	O antigen-negative mutant of strain 8624	–	Bilge et al. (1996)

*^a^+, susceptible to Φ241; –, not susceptible to Φ241*.

*^b^ATCC, American Type Culture Collection*.

### BRINE SAMPLE COLLECTION AND TREATMENT

To isolate *E. coli* O157:H7-specific phages, brine samples (40 ml each) were taken from seven industrial cucumber fermentation tanks (capacity: 32,000 l) from a commercial processing plant. The tanks contained approximately 55% pickling cucumbers in 5 to 8% recycled NaCl brine, prepared essentially as described by Breidt et al. (2013). These samples were taken during the fermentation (3–5 days after the tanks were packed and brined). Samples were transported to the laboratory at ambient temperature (∼23°C), stored at 4°C, and processed within 24 h. The pH of each brine sample was measured and adjusted to around 6.4 with 5 M NaOH. The pH-adjusted brine samples were then centrifuged (5,000 × *g* for 10 min). The supernatants were filtered through syringe filters (0.45 μm pore size) to remove cellular materials and solid particles. The filtrates were stored at 4°C until used as potential phage source for phage isolation.

### PHAGE ISOLATION

Ten *E. coli* O157:H7 strains (shown in bold text, **Table 1**) were used as potential hosts for phage isolation. Overnight cultures of these O157 strains (∼10^9^ CFU/ml) were prepared in TSB. A 96-well microplate was used to enrich phages potentially present in the filtered brines. Each well of the microplate contained 200 μl of TSB, 5 μl of one of the 10 *E. coli* O157:H7 strains and 45 μl of one of the eight filtered brines, so the eight wells in the same column received the same O157:H7 strain. The first 10 wells in the same row received the same filtered brine. After incubation at 37°C for 20 h, the microplate was centrifuged (SH-3000 rotor, RC-5B centrifuge, Sorvall, Newtown, CT, USA) at 4,000 rpm, 4°C for 20 min. The supernatant (lysate) in each well was collected and used in spot tests to detect the presence of phages. Each spot test was performed by adding 10 μl of a phage lysate onto a lawn of *E. coli* O157:H7 in a soft agar overlay on a TSA plate. After overnight incubation at 37°C, the plates were checked for a zone of bacterial lysis.

### PHAGE PURIFICATION AND CONCENTRATION

Phage from a positive spot-test plate was purified and concentrated using the methods described by Lu et al. (2003) with minor modification. Briefly, an isolated single plaque was picked and propagated against its natural host in TSB at 37°C. After two runs of plaque purification, the phage lysate was prepared and then centrifuged at 5,000 × *g* for 10 min. The supernatant was filtered through bottle-top filter (0.45 μm pore size). The filtered high titer phage stock (typically ca. 10^10^ PFU/ml) was stored at 4°C. To further purify and concentrate the phage, a portion of the phage stock were treated with DNase I and RNase A, and then concentrated by PEG precipitation. The concentrated phage was further purified by CsCl step density gradient ultracentrifugation at 600,000 × *g* for 6 h at 4°C followed by dialysis as described by Lu et al. (2003). The ultracentrifuge-purified phage was used for electron microscopy analysis, SDS-PAGE, and DNA extraction.

### ELECTRON MICROSCOPY

Phage samples were negatively stained with 2% (w/v) aqueous uranyl acetate (pH 4) on carbon-coated grids and examined by transmission electron microscopy (JEM 1200EX TEM, JEOL) at an accelerating voltage of 80 kV. Electron micrographs were taken at a magnification of 50,000× (Center for Electron Microscopy, North Carolina State University, Raleigh, NC, USA).

### ONE-STEP GROWTH KINETICS

One step growth experiments were carried out based on the method described by Leuschner et al. (1993) and Foschino et al. (1995) with some modifications. Briefly, the experiment started at a multiplicity of infection (MOI) of 0.01 in a 15-ml tube containing the phage (approximately 1 × 10^6^ PFU/ml) and its natural host O157:H7 strain B0241 in 10 ml TSB. After incubation in a water bath at 37°C for 10 min (to allow phage adsorption), the tube was centrifuged at 13,000 × *g* for 30 s. The supernatant was removed and subjected to plaque assay to determine the titer of the un-absorbed phage. The pellet containing (partially) infected cells was immediately re-suspended in 10 ml of pre-warmed TSB. After taking the first sample, the tube was returned to the water bath (37°C). A sample (100 μl) was collected every 5 min (up to 60 min). Each sample was immediately diluted and subjected to plaque assay. All assays were carried out in triplicate. The experiment was repeated three times. Latent period was defined as the time interval between the end of the adsorption and the beginning of the first burst, as indicated by the initial rise in phage titer (Ellis and Delbruck, 1939; Adams, 1959). Burst size was calculated as the ratio of the final number of liberated phage particles to the initial number of infected bacterial cells during the latent period (Adams, 1959).

### HOST RANGE

Phage Φ241 was the only phage isolated from one of the seven samples. The host range of Φ241 was determined by spot tests against 46 *E. coli* O157:H7 strains (**Table 1**) and 18 non-O157 strains (**Table 2**) on TSA. In each test, 10 μl of high titer phage stock (10^10^ PFU/ml) was used to spot a bacterial lawn of a strain on a plate. Each test was done in duplicate. The O antigen-negative mutants of *E. coli* O157:H7 and their parent strains (**Table 3**) were also tested using the agar overlay method.

### PHAGE STRUCTURAL PROTEINS

The phage structural proteins were analyzed using the method previously described by Lu et al. (2003) with some modifications. Briefly, the ultracentrifuge-purified phage particles were mixed with SDS-PAGE sample buffer and then heated in a boiling water bath for 10 min. The boiled sample was loaded onto a NuPAGE precast gradient minigel (4–12% Bis-Tris, Invitrogen Corporation, Carlsbad, CA, USA). Electrophoresis was carried out at 75 V for 2 h. Pre-stained protein standard (Invitrogen) was used to estimate the molecular weights of the proteins. The gel was stained with SimplyBlue SafeStain (Invitrogen).

### PHAGE DNA EXTRACTION AND RESTRICTION

Phage DNA was prepared from the concentrated lysate using the phenol–chloroform extraction method as described by Lu et al. (2003), and digested with restriction endonucleases (AluI, BamHI, ClaI, EcoRI, EcoRV, HindIII, MspI, SwaI, and XbaI; New England BioLabs, Beverly, MA, USA) according to the manufacturer’s instructions. The resulting DNA fragments were separated on the 1% agarose gel containing 0.001% SYBR Safe DNA gel stain (Invitrogen) by gel electrophoresis in Tris-borate-EDTA buffer at 70 V for 2 h. The 1 kb DNA ladder (Promega, Madison, WI, USA) was used to estimate the size of the digested phage DNA.

### PHAGE INFECTION

The lytic activity of phage Φ241 against host *E. coli* O157:H7 B0241 was investigated in TSB medium at three different MOIs. A bacterial overnight culture was diluted with TSB to a concentration of ca. 9 × 10^6^ CFU/ml. Ten milliliter of the diluted bacterial culture was then transferred into each of the four 15-ml tubes. One of these tubes served as a control. To each of other three tubes, a high titer phage stock (2.8 × 10^10^ PFU/ml) was added to achieve an initial MOI of 10, 3, or 0.3, respectively. The four tubes were incubated statically at 37°C. Samples were taken from each tube at 60-min intervals for a 12-h period. After serial dilution, each sample was plated onto TSA plates using a spiral autoplater (Model 4000, Spiral Biotech, Bethesda, MD, USA). The plates were incubated at 37°C overnight. The colonies on each plate were enumerated using Q-Count system (Model 510, Spiral Biotech, Norwood, MA, USA). The experiment was repeated two more times.

### STATISTICAL ANALYSIS

Differences in bacterial cell concentration between various grouping of MOIs were analyzed by using one-way analysis of variance (ANOVA) and Tukey’s multiple comparison.

## RESULTS AND DISCUSSION

### ISOLATION OF PHAGE Φ241

Seven brine samples from 32,000-l cucumber fermentation tanks (all from the same commercial plant) were enriched for phage isolation. One sample was found to contain a phage that infects *E. coli* O157:H7. The phage-containing sample was taken from a tank 3 days after the tank was packed with size 2A cucumbers (∼27–32 mm in diameter). The pH and the salt (NaCl) concentration of the sample were 3.7 and 5%, respectively. In contrast, the pH and salt concentration of the samples from other six tanks were in the range of 3.42–3.92, and 6 to 8%, respectively. The higher salinity in these six tanks may greatly inhibit phages, which may explain why no O157:H7 phages were isolated from them. The isolated O157:H7 phage was designated Φ241. The presence of phage Φ241 specifically active against *E*. *coli* O157:H7 in an early stage of the commercial cucumber fermentation indicates that the host strain(s) may be present as well. The most likely source for *E*. *coli* O157:H7 in the commercial fermentation would be the fresh cucumbers. Application of animal waste as fertilizer and irrigation of crops with waste water have been recognized as important routes through which *E*. *coli* O157:H7 can contaminate fresh vegetables during primary production (Ongeng et al., 2013). However, we are unaware of any reports of disease outbreaks caused by vegetative pathogens from fermented vegetables. Previous research has shown that *E*. *coli* O157:H7 will be killed during fermentation of cucumbers in a pH and time dependent manner (Breidt and Caldwell, 2011).

The isolated phage Φ241 forms small (ca. 1 mm in diameter) plaques on the lawn of its natural host, *E. coli* O157:H7 strain B0241 which contains *stx*2 gene and was originally isolated from bovine carcass (**Table 1**). The concentration of high-titer phage stock (ca. 10^10^ PFU/ml) remained unchanged during 2 years of storage at a refrigeration temperature, indicating that the phage is very stable.

### MORPHOLOGY

**FIGURE 1:**
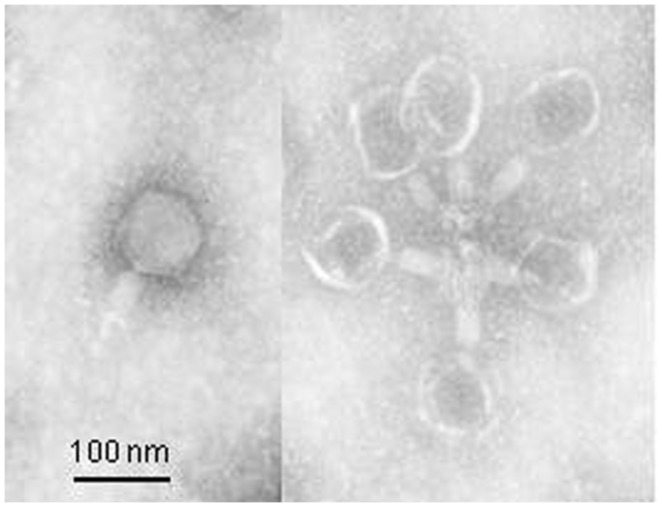
**Transmission electron micrograph of phage Φ241 negatively stained with 2% uranyl acetate (pH 4).** Scale bar, 100 nm.

The electron micrograph (**Figure 1**) showed that phage Φ241 has an icosahedral head (about 80 nm in diameter) and a contractile tail (ca. 33 nm long in the contracted state) with a base plate and several tail fibers. The overall morphology of Φ241 indicates that it is a T4-like phage, belonging to the *Myoviridae* family of the *Caudovirales* order. Interestingly, several phage particles appeared to cluster together through the tail fibers (**Figure 1**). The base plate and tail fibers are usually involved in the host cell recognition and receptor-binding by many tailed phages (Riede, 1987; Leiman et al., 2004; Bartual et al., 2010; Garcia-Doval and van Raaij, 2012).

### ONE-STEP GROWTH KINETICS

**FIGURE 2:**
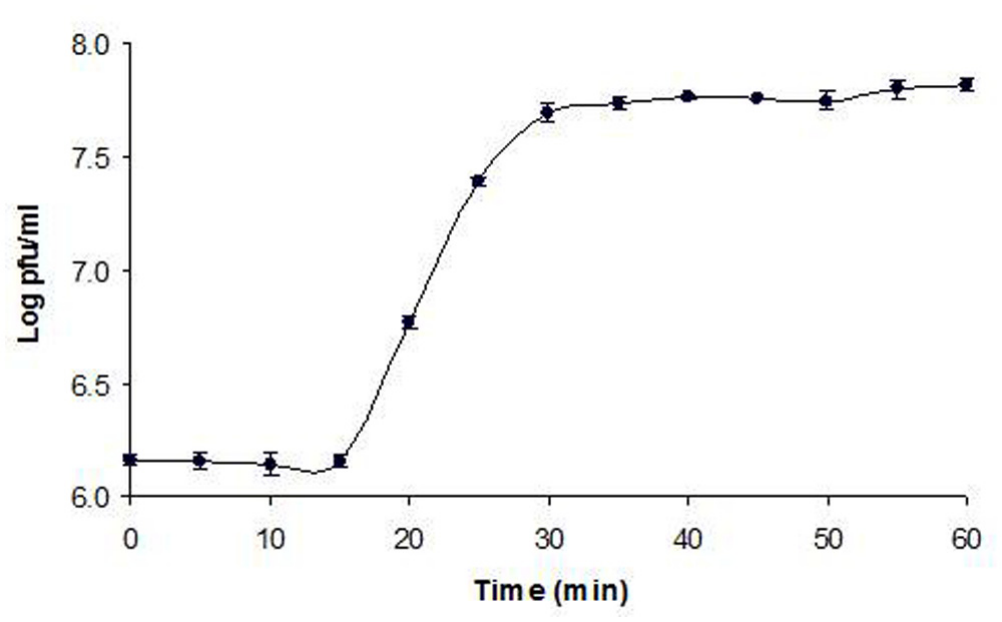
**1-step growth curve of phage Φ241 infecting *Escherichia coli* O157:H7 at MOI 0.01 in TSB medium at 37°C.** The latent period is 15 min. The error bars indicate standard deviations.

**Figure 2** shows the one-step growth of phage Φ241. The latent period was only 15 min (excluding 10 min for adsorption), which is shorter than the typical latent periods (21–120 min) for most *Myoviridae* phages. A short latent period allows phage Φ241 to replicate faster than most *Myoviridae* phages. The average burst size of Φ241 was about 53 phage particles per infected cell, which is in the range of 50–100 PFU/cell for many *Myoviridae* phages (Foschino et al., 1995; Chang et al., 2005; Raya et al., 2006; Bao et al., 2011; Park et al., 2012). A few *Myoviridae* phages have very large burst sizes. The burst size of phage PhaxI (another O157:H7 phage) is 420 PFU per cell (Shahrbabak et al., 2013). A phage with both a short latent period (15 min or less) and a large burst size (>50 PFU/cell) may have a selective advantage over competing phages, resulting in very high lytic activity (Park et al., 2012).

### HOST RANGE

A total of 69 *E. coli* strains from various sources (**Tables 1**–**3**) were tested to determine the host range of phage Φ241. The phage was able to lyse all 46 O157:H7 strains (**Table 1**), but none of the 18 non-O157 strains (**Table 2**) including O104:H7 strain which has the same H antigen as that of O157:H7. *E. coli* O104:H7 was originally isolated from ground beef (Bosilevac and Koohmaraie, 2011). It is also Shiga toxin-producing strain containing two uncommon Shiga toxin gene variants, *stx*_1c_ and *stx*_2c_ (Bosilevac and Koohmaraie, 2011). The data suggested that the phage is O157 antigen specific, and H7 antigen may not be involved in the host recognition and binding. Phage infection requires specific receptors on bacterial cells. The common receptors on *E. coli* include O antigen of lipopolysaccharide (LPS), outer membrane proteins, pili, fimbriae, and flagella (H) antigen (Topley and Wilson, 1990; Bokete et al., 1997). Many cell wall receptors can be shared by different bacterial strains and serotypes (Topley and Wilson, 1990). To confirm that O157 antigen (not H7 antigen) serves as the receptor during Φ241 adsorption, two previously described O antigen-negative mutants (43895Δ*per* and F12), one *per*-complemented mutant (43895Δ*per*Comp), and two *E. coli* O157:H7 parent strains (ATCC 43895 and 8624) were tested for their susceptibility to Φ241 infection (**Table 3**). The mutant 43895Δ*per* was generated by deletion of a putative perosamine synthetase gene (*per*) in the *rfb* gene cluster (Sheng et al., 2008). The mutant F12 was created by transposon insertion of Tn*phoA* in the *per* gene (Bilge et al., 1996). Deletion of *per* gene or insertion in *per* gene resulted in a mutant lacking the O antigen. The Δ*per* mutant (43895Δ*per*) also lacked H7 antigen, but the transposon insertion mutant (F12) still expressed the H7 antigen. The *per*-complemented mutant (43895Δ*per*Comp) was constructed by cloning *per* in the *E. coli* vector pCRII and transforming pCRII::*per* into the mutant to restore O157 antigenicity (Sheng et al., 2008). **Table 3** showed that phage Φ241 lysed the two O157:H7 parent strains (ATCC 43895 and 8624) which had the full-length O157 antigen, and the *per*-complemented strain (43895Δ*per*Comp) which was able to express O157 antigen. But the phage did not lyse the two O157 antigen-negative mutants, 43895Δ*per* (also lacking H7 antigen) and F12 (still having H7 antigen). These results supported our hypothesis that O157 antigen is required for the infection by phage Φ241, and strains lacking O157 antigen were resistant to the phage infection, regardless of the presence or absence of H7 antigen in the strains. Similar observations have been reported for other O157-specific phages. Kudva et al. (1999) studied three O157-specific phages isolated from bovine and ovine fecal samples. They found that the three phages lysed all of the eight tested *E. coli* O157 strains including the strain 8624 and did not lyse non-O157 *E. coli* strains, or O157-negative mutants including F12. In addition, the three phages did not lyse the complement of the O157-deficient mutant, F12(pF12), which produces a truncated O157 LPS (Kudva et al., 1999). They found that phage infection and plaque formation were influenced by the structure of the host cell O157 LPS. Strains that did not express the O157 antigen or expressed a truncated LPS were not susceptible to plaque formation or lysis by phage. Interestingly, strains that expressed abundant mid-range-molecular-weight LPS were lysed in broth media but did not support plaque formation. They explained that in broth media, the excess mid-range-molecular-weight LPS can diffuse from cells into the broth. But on soft agar, those molecules may accumulate around cells, thereby preventing phage attachment (Kudva et al., 1999). An appropriate length of the O side chains and an optimal LPS concentration may be necessary to make the receptor available for phage interaction and/or to allow irreversible phage binding (Calendar, 1988). The high specificity of phage Φ241 for O157 antigen makes it an ideal biocontrol agent of *E. coli* O157:H7 without disrupting the beneficial bacteria such as probiotics in foods, normal flora in humans, or other microflora in cattle.

### STRUCTURAL PROTEINS

**FIGURE 3:**
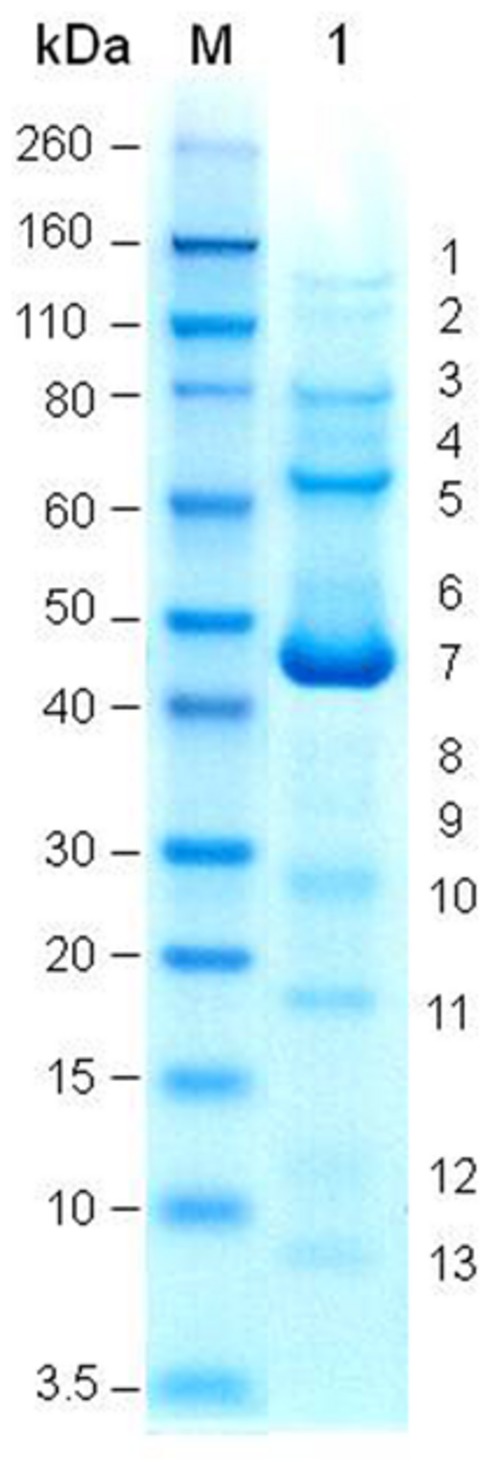
**SDS-PAGE of Φ241 structural proteins.** Lane M: molecular weight (MW) standard; lane 1: Φ241. The MWs of protein bands in the standard are indicated on the left.

SDS-PAGE gel revealed at least 13 protein bands from Φ241 (**Figure 3**), indicating that the phage contains many types of structural proteins. Four of the protein bands are in the molecular weight (MW) range of 26 to 50 kDa. These include three weak bands and one strong band (band 7 in **Figure 3**, MW≈44 kDa). In fact, this strong band is the strongest one among all bands, indicating that the protein in this band is the most abundant protein. In many tailed phages, the most abundant proteins are usually identified as the major head proteins (Santos et al., 2011). The MWs of major head proteins generally fall within the range of 26–50 kDa. For example, the sequence-predicted MWs of the major capsid protein in *Lactobacillus plantarum* phage ΦJL-1 (Lu et al., 2005), O157:H7 phage PhaxI (Shahrbabak et al., 2013), *Pseudomonas aeruginosa* phages LKA1 and LKD16 (Ceyssens et al., 2006), *Salmonella enterica* phage PVP-SE1 (Santos et al., 2011) are 30.4, 48.0, 36.7, 37.7, and 38.5 kDa, respectively. Since SDS-PAGE analysis can only reveal a very limited number of structural proteins, genomic studies are needed in order to better understand phage structural proteins and their functions.

### DNA RESTRICTION

**FIGURE 4:**
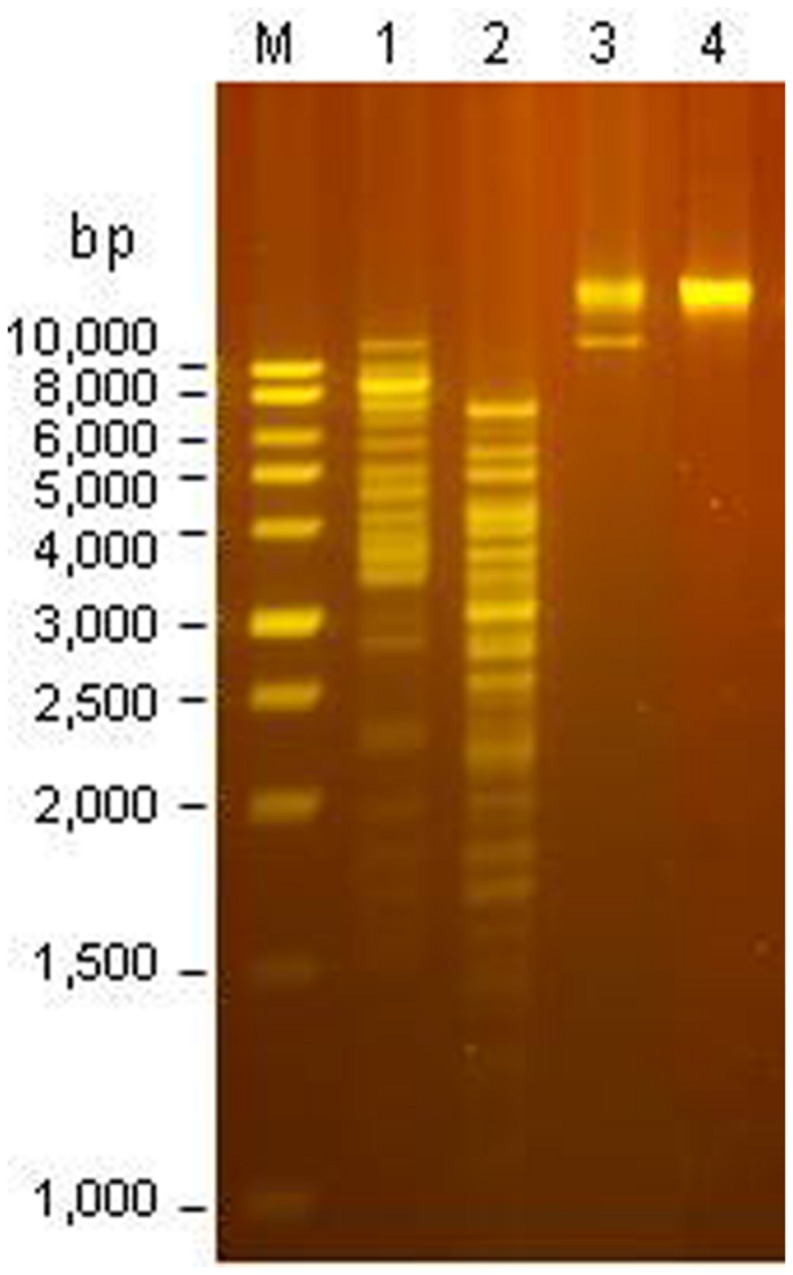
**Restriction analysis of the DNA from Φ241.** Lane M: 1-kb ladder; Lane 1: digestion by AluI; lane 2: digestion by MspI; lane 3: digestion by SwaI; lane 4: undigested DNA.

The Φ241 genome could be digested by rare-cutters, AluI, MspI, and SwaI (**Figure 4**). Restriction by AluI or MspI generated more than 15 bands on agarose gel while restriction by SwaI only generated a single band with a high MW. The phage genome could not be digested by many commonly used restriction endonucleases such as BamHI, ClaI, EcoRI, EcoRV, HindIII, and XbaI. Similar phenomenon was also observed for other O157:H7-specific phages. Shahrbabak et al. (2013) reported that the genome of phage PhaxI was resistant to eight tested restriction endonucleases including BamHI, EcoRI, EcoRV, HindIII, and a few others (Shahrbabak et al., 2013). The resistance suggested the presence of modification such as methylation and glycosylation in the phage DNA, allowing the phage to evade the restriction by the host enzymes (Bickle and Kruger, 1993; Nechaev and Severinov, 2008; Vasu and Nagaraja, 2013). Sequence analysis may provide insight into the anti-restriction modification system in phage genome.

### PHAGE INFECTION

**FIGURE 5:**
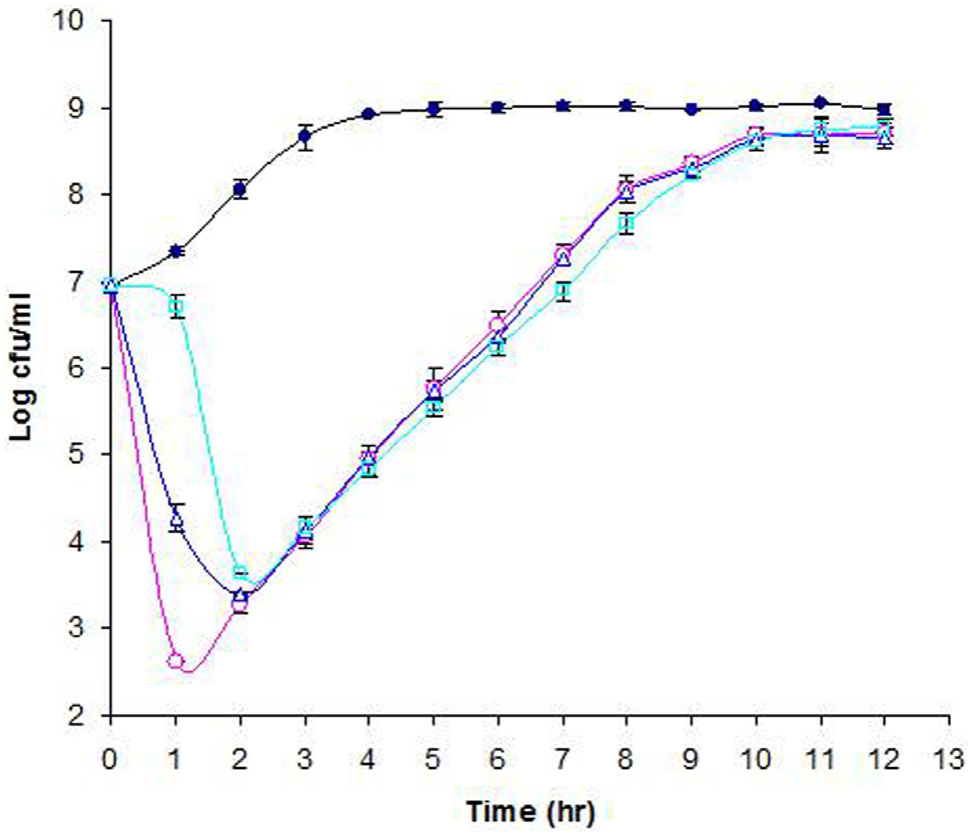
**Lytic activity of phage Φ241 against *E. coli* O157:H7 in TSB medium at MOI 10 (o), 3 (Δ), or 0.3 (□).** The control (∙) contains only *E. coli* O157:H7. All cultures were incubated at 37°C. The error bars indicate standard deviations in triplicate experiments.

The lytic activity of phage Φ241 against its natural host *E. coli* O157:H7 B0241 was investigated at three different MOIs. **Figure 5** shows the growth curves of phage-free and phage-infected cultures in TSB medium at 37°C. The phage-free culture (the control culture) grew steadily during the first 4 h of incubation. After 4 h, the control culture entered the stationary phase and remained unchanged (**Figure 5**). In contrast, the phage infection at the MOI of 3 or 10 caused a rapid cell lysis within 1 h, resulting in 3- or 4.5-log decrease in the cell concentration. Such a high lytic activity within 1 h may be attributed in part to the short latent period (15 min) of the phage. During the second hour, the cell concentration of the culture with a MOI of 3 continued to decrease while the cell concentration of the culture with the MOI of 10 started to increase. In contrast, infection at the MOI of 0.3 initially caused a slow cell lysis (less than 0.5-log reduction) during the first hour, but a rapid cell lysis (3-log reduction) during the second hour. The data from statistical analysis showed that at 1 h after phage infection the cell concentrations from different MOIs were statistically different (P < 0.05) and every cell concentration was different from all other cell concentrations (α = 0.05). At 2 h after phage infection the cell concentration from the MOI of 0.3 was statistically different from all other cell concentrations while the cell concentrations from the initial MOIs of 3 and 10 were not statistically different. Similar rapid cell lysis caused by Φ241 in cucumber juice was also observed (preliminary data not shown). Kudva et al. (1999) evaluated the lytic activity of three O157-specific phages in Luria-Bertani medium supplemented with 5 mM MgSO_4_ at 37°C. They reported that the significant (>4 log) decrease in *E. coli* O157:H7 concentration caused by those phages individually or in cocktail required much higher MOI (10^3^ PFU/CFU) and much longer incubation time (8 h) compared with those in our study. **Figure 5** showed that the cultures with an initial MOI of 3 or 0.3 started to grow after 2 h. Interestingly, after 3 h of infection, all three phage-infected cultures, regardless of the initial MOI, reached the same cell concentration (10^4^ CFU/ml), which was 4.5-log lower than that of the control and 3-log lower than the initial cell concentration. As the incubation continued, the three cultures continued to grow at a similar rate, gradually approaching to the cell concentration of the control. After 12 h of infection, the phage titers in the cultures at the initial MOI of 10, 3, and 0.3 reached 4 × 10^9^, 5 × 10^9^, and 1.6 × 10^10^ PFU/ml, respectively. Apparently, the culture started with the lowest initial MOI (0.3) contained the highest phage titer (1.6 × 10^10^ PFU/ml) at the end of incubation.

The growth of phage-infected cultures after 1 or 2 h of infection indicated that phage-resistant mutants had emerged. The emergence of phage-resistant mutants during phage infection has been reported by many other studies (Kudva et al., 1999; O’Flynn et al., 2004; Park et al., 2012; Tomat et al., 2013). Phage resistance may result from mutation that alters cell surface receptors, restriction modification, or abortive infection associated with the presence of clustered regularly interspaced short palindromic repeats (CRISPRs) in the bacterial genome (Hill, 1993; Hashemolhosseini et al., 1994; Allison and Klaenhammer, 1998; Barrangou et al., 2007). A few studies found that certain phage resistant mutants of *E. coli* O157:H7 had altered OmpC expression or lost OmpC, suggesting the involvement of the major outer membrane protein in phage attachment (Yu et al., 2000; Morita et al., 2002; Mizoguchi et al., 2003). Some studies found that cell morphology and colony morphology of phage-resistant mutants differed greatly from those of the parent *E. coli* O157:H7 strains (Mizoguchi et al., 2003; O’Flynn et al., 2004). Phage-resistant mutant cells appeared coccoid and smaller. As a result, phage-resistant culture could not reach the same turbidity as that of the parent strain culture (O’Flynn et al., 2004). The frequency of phage-resistant mutation is generally around 10^-6^ CFU for *E. coli* O157:H7 (O’Flynn et al., 2004; Park et al., 2012; Tomat et al., 2013). With such a low mutation frequency and the low level of *E. coli* O157:H7 typically encountered in foods, phage resistance should not hinder the use of phages as biocontrol agents against the pathogenic bacteria (O’Flynn et al., 2004; Tanji et al., 2004). Some studies explored the potential of using a phage cocktail to minimize the development of phage resistant mutants on meats and other foods (O’Flynn et al., 2004; Tanji et al., 2004; Carter et al., 2012; Tomat et al., 2013). Using a phage cocktail containing different phages against the same bacterial species can decrease the likelihood of selecting phage-resistant mutants. Because different phages may attach to different receptors on the host, mutations in one phage receptor gene may not alter the mutant’s susceptibility to another phage that attaches to a different receptor on the bacterial cells (Tanji et al., 2004).

In conclusion, phage Φ241 is highly specific for *E. coli* O157:H7 and very stable when stored at high titers at refrigeration temperature. The phage causes rapid cell lysis, and tolerates both low pH and high salinity. These features indicate that the phage has a high potential as an effective biocontrol agent of *E. coli* O157:H7 in foods. Trials are under way to evaluate the efficacy of the phage to control *E. coli* O157:H7 in various foods including acidic and/or salty foods. To our knowledge, this is the first report on the *E. coli* O157:H7 phage isolated from low pH and high salinity environment.

## Conflict of Interest Statement

The authors declare that the research was conducted in the absence of any commercial or financial relationships that could be construed as a potential conflict of interest.
